# Autonomic Receptor Autoantibodies in Complex Regional Pain Syndrome and Other Chronic Pain Conditions: A Cross-Sectional Analysis

**DOI:** 10.3390/biomedicines14040945

**Published:** 2026-04-21

**Authors:** Daniël P. C. van der Spek, Renée H. Hoffenkamp, Frank J. P. M. Huygen, Krishna D. Bharwani, Niels Eijkelkamp, Maaike Dirckx

**Affiliations:** 1 Department of Anesthesiology, Center for Pain Medicine, Erasmus MC University Medical Center, 3015 GD Rotterdam, The Netherlands; 2 Center for Translational Immunology, University Medical Center Utrecht, 3584 CX Utrecht, The Netherlands

**Keywords:** complex regional pain syndrome, chronic pain, autoantibodies, G-protein-coupled receptors, autoimmunity

## Abstract

**Objectives**: Complex regional pain syndrome (CRPS) is a heterogeneous pain disorder with incompletely understood immunoinflammatory features. This study investigated whether autonomic receptor autoantibodies differentiate CRPS from other chronic pain conditions and healthy controls. **Methods**: We conducted a cross-sectional analysis of serum samples from patients referred with suspected CRPS. Patients were subsequently classified as having either CRPS or another chronic pain condition, based on the Budapest criteria. Healthy controls were included for comparison. Serum levels of autoantibodies targeting the muscarinic M2 receptor (M2R), β1-adrenergic receptor (β1AR), and the β2-adrenergic receptor (β2AR) were assessed using enzyme-linked immunosorbent assay. All analyses were performed blinded to group assignment. **Results**: Seventy participants were analyzed (CRPS = 22, other chronic pain = 25, healthy controls = 23). M2R autoantibody levels were higher in both CRPS and other chronic pain compared with healthy controls (mean difference [MD] = 0.37, 95%CI 0.22–0.51; and MD = 0.31 95%CI 0.19–0.44, respectively). β2AR levels were higher in other chronic pain compared with healthy controls (MD = 0.29, 95%CI 0.04–0.54), whereas no significant difference was observed in CRPS (MD = 0.21 95%CI −0.01–0.42). No meaningful differences were observed between CRPS and other chronic pain for any receptor. β1AR levels did not differ between groups. Seropositivity for any autoantibody was 55% in CRPS, 44% in other chronic pain, and 22% in healthy controls. **Conclusions**: Elevated autonomic receptor autoantibody levels were observed across chronic pain conditions but were not specific for CRPS.

## 1. Introduction

Complex regional pain syndrome (CRPS) lacks objective biological markers, complicating early recognition and contributing to prolonged disability and reduced quality of life [1,2]. CRPS is commonly triggered by trauma and presents with symptoms that are disproportionate in severity and duration to the initial injury. The disease has a dynamic course, with symptoms fluctuating across and within patients over time [3]. The clinical picture of CRPS is thought to arise from multiple mechanisms, with an aberrant immunoinflammatory response as the initiating trigger, followed by vasomotor dysfunction and alterations in the peripheral and central nervous system, but its exact pathophysiology is not yet fully understood [4,5]. Consequently, CRPS remains a clinical diagnosis based on the International Association for the Study of Pain diagnostic criteria, commonly referred to as the “Budapest criteria” [3]. These criteria have recently been refined in the Valencia consensus-based adaptation [6]. Management is likewise challenging, as there is no one-size-fits-all approach [7]. This clinical and biological heterogeneity has complicated the efforts to define disease-specific mechanisms in CRPS.

Given the proposed immunoinflammatory trigger [4,5,8,9,10], immune-related candidates are receiving particular attention as potential biological markers [11]. Elevated levels of antinuclear antibodies (ANAs) and other autoantibodies have been reported, suggesting an autoimmune or autoinflammatory predisposition to the disease [10,12,13,14]. Subsequently, specific autoantibodies targeting G protein-coupled receptors (GPCRs), including the α-1A-adrenergic receptor (α1A-AR), the β2-adrenergic receptor (β2AR), and the muscarinic M2 receptor (M2R), have been identified in the serum of patients [10,15,16]. Functional effects of patient-derived IgG have been demonstrated in animal models, where transfer of human IgG induced CRPS-like features [17,18,19]. However, the extent to which these experimental observations translate to human disease expression remains uncertain, and independent replication in clinical cohorts has been limited. Consequently, it remains unclear whether these reported autoantibody alterations are specific to CRPS or a more general reflection of chronic pain. The aim of this study was to quantify and compare the serum levels of autonomic receptor autoantibodies targeting the M2R, β1-adrenergic receptor (β1AR), and β2AR in patients with confirmed CRPS, patients with suspected CRPS who were subsequently diagnosed with other chronic pain conditions, and healthy controls.

## 2. Materials and Methods

### 2.1. Study Design

We conducted a cross-sectional, post hoc analysis of leftover serum samples that had been prospectively collected between March 2018 and August 2019 as part of the ImPaCt study [20]. In the original single-center ImPaCt study conducted at the Erasmus MC outpatient pain clinic, adults referred with a clinical suspicion of CRPS were prospectively enrolled and classified using the Budapest criteria [3]. Participants who met the criteria comprised the CRPS group, whereas those who did not meet the criteria received an alternative diagnosis and comprised the other chronic pain group (e.g., myofascial pain syndrome, neuropathic pain or inflammation). Eligible participants were ImPaCt enrollees who had given consent for future use of residual materials. All eligible participants with stored serum were included, and their clinical parameters from the ImPaCt study were used. Healthy controls were included for comparison, consisting of volunteers participating in research at Erasmus MC. Patients and healthy controls with a history of autoimmune disease were excluded. To reduce measurement and classification bias, samples were pseudonymized, and investigators performing the assays were blinded to these pre-existing group assignments. All samples were processed using identical reagents and procedures. The autonomic receptor autoantibody analyses were performed in the immunology laboratory of the University Medical Center Utrecht. The study reporting adheres to the STROBE guideline for cross-sectional studies [21].

### 2.2. Outcomes

The primary outcome was the serum level of autonomic receptor autoantibodies targeting the M2R, β1AR, and β2AR, compared between patients with CRPS, patients with other chronic pain conditions, and healthy controls.

Secondary outcomes included seropositivity for these autoantibodies, defined relative to healthy controls. In addition, ANA seropositivity was assessed to replicate previous findings reported by our group [14]. As an exploratory outcome, we assessed the correlation between autoantibody levels and the CRPS severity score within the CRPS group.

### 2.3. Materials

Peptides corresponding to the GPCRs’ second extracellular loop of M2R, β1AR, and β2AR were synthesized by GenScript (Piscataway, NJ, USA). The sequences were RTVEDGECYIQFFSNAAVTFGT for M2R (residues 170 to 191), HWWRAESDEARRCYNDPKCCDFVTNR for β1AR (residues 197 to 222), and HWYRATHQEAINCYANETCCDFFTNQ for β2AR (residues 172 to 197). Peroxidase-conjugated AffiniPure Goat Anti-Human IgG Fc gamma fragment specific 109-035-098 (anti-human IgG-HRP) from Jackson ImmunoResearch (West Grove, PA, USA) was used as the secondary antibody together with a ready-to-use commercial one-component 3,3′,5,5′-tetramethylbenzidine (TMB) substrate from BioFX (Owing Mills, MD, USA; LOT: TMBWY29). The coating buffer was prepared by dissolving Na_2_CO_3_ (EMSURE, Merck KGaA, Darmstadt, Germany; LOT: 1063920500) in distilled water (pH = 10.3). The wash buffer was phosphate-buffered saline from Thermo Fisher Scientific (Waltham, MA, USA; LOT: RNBN0239) with 0.05% Tween 20 (Fisher bioreagents, Pittsburgh, PA, USA; BP337-500; LOT: 225151). The blocking buffer was 1% bovine serum albumin Fraction V from Sigma Aldrich (St. Louis, MO, USA; LOT: 70907140) in wash buffer.

### 2.4. Enzyme-Linked Immunosorbent Assay

We assessed serum autoantibodies using an enzyme-linked immunosorbent assay (ELISA) adapted from protocols by Galloway and Kohr [10,22]. The assay was optimized and standardized using human serum samples with known high titers of autoantibodies against β1AR, β2AR, and M2R to ensure consistent assay performance. The workflow followed five steps in order: coating, blocking, primary binding, secondary binding, and quantification. Plates were washed 3 times with wash buffer between each step.

F96 MaxiSorp microplates from Thermo Fisher Scientific (LOT: 184540) were coated overnight at 4 °C with 100 µL of coating buffer containing peptide at 1 µg/mL corresponding to the second extracellular loop of β1AR, β2AR, or M2R. The next day, plates were blocked with 150 µL of blocking buffer for 1 h at 37 °C. After blocking, serum samples diluted 1 to 100 in blocking buffer were added at 100 µL per well and incubated for 2 h at 4 °C to allow binding to second extracellular loop epitopes. Plates were then incubated with anti-human IgG-HRP at 1 to 20,000 in blocking buffer for 1 h at 37 °C. Next, after washing 50 µL of TMB substrate was added at room temperature, followed by 50 µL of stop solution after 20 min. Optical density was read at 450 nm within 5 min on a Clariostar plate reader.

For β2AR, two methods were used. In addition to the in-house ELISA described above, a commercially available ELISA kit from CellTrend GmbH (Luckenwalde, Germany; #12700) was used according to the manufacturer’s protocol. Because the CellTrend assay uses the full length β2AR, it was included as a complementary test to assess the robustness of findings obtained with the in-house ELISA directed against the second extracellular loop domain.

### 2.5. Laboratory Testing

ANA tests were performed in the diagnostic laboratory of the University Medical Center Utrecht. The 95th percentile of blood donors was used as the cut-off for a positive serum ANA [23]. Based on previous data showing 4% ANA positivity in a healthy cohort (n = 90, *p* < 0.001) [14], ANA levels were not determined for the healthy controls in this study.

### 2.6. Statistical Analysis

All analyses were performed using R (version 4.4.1). Data distributions were assessed visually using histograms and residual plots and using the Shapiro–Wilk test. Continuous data are presented as mean with standard deviation (SD) for approximately normally distributed variables and as median with interquartile ranges (IQR) otherwise. Autoantibody serum levels (β1AR, β2AR, or M2R), were compared between groups using Welch’s *t*-test for pairwise comparisons. Effect sizes are reported as mean difference (MD) with 95% confidence intervals (CI). Receiver operating characteristic (ROC) analyses were performed to explore threshold-independent individual-level discrimination of autoantibody serum levels. For these exploratory analyses, patients with CRPS and other chronic pain were combined into a single chronic pain group and compared with healthy controls. ROC analyses were not used to define diagnostic cut-offs. Within the CRPS group, exploratory associations between autoantibody levels and CRPS severity score (CSS) [24] were assessed using Spearman’s rank correlation coefficient (ρ).

Seropositivity for autonomic autoantibodies was defined using a conservative cut-off based on the mean +2 SD (in optical density [OD]) of healthy controls for each receptor peptide, as previously described by Galloway et al. [22]. Extreme outliers (OD > 2) were excluded from cut-off determination but retained in all analyses and graphical displays. ANA results were regarded positive when the read-out was at least weak positive. Group differences in seropositivity were assessed using chi-squared or Fisher’s exact tests, and effect sizes are reported as odds ratios (ORs) with 95% CI. For assay validation, a sensitivity analysis was performed using a commercially available assay (CellTrend). Autoantibody values from the CellTrend assay were expressed in arbitrary units extrapolated from a standard curve generated using five calibrators (2.5–40 U/mL), and positivity was defined according to the manufacturer’s protocol. *p*-values < 0.05 (two-sided) were considered statistically significant.

## 3. Results

### 3.1. Baseline Characteristics

Of the 52 participants with a suspicion of CRPS in one limb in the original study, residual serum was unavailable for 5, leaving 47 for analysis. Of these 47 individuals, 22 received a CRPS diagnosis and 25 had other chronic pain conditions (myofascial pain syndrome, n = 14; neuropathic pain, n = 6; inflammation, n = 2; unknown/unclear etiology, n = 3). The CRPS group had a median age of 43 years (IQR 30–54) and a median disease duration of 26 months (IQR 16–74). Baseline characteristics did not differ significantly between the CRPS and other chronic pain group, and neither group differed from the 23 healthy controls in age or sex (Table 1).

### 3.2. Autonomic Receptor Antibodies and ANAs

**Figure 1:**
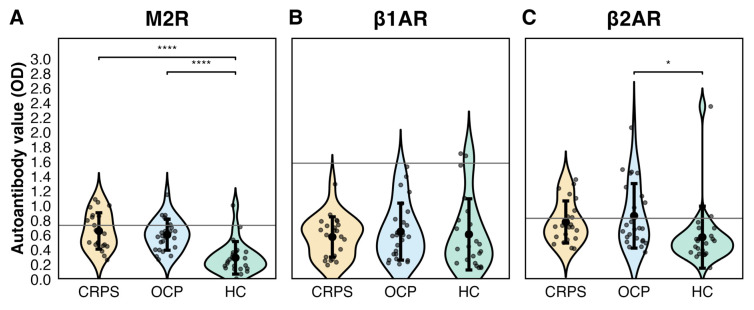
Autoantibody levels against the second extracellular loop of the (**A**) M2R, (**B**) β1AR, and (**C**) β2AR in serum from healthy controls, patients with other chronic pain (OCP), and patients with CRPS. OCP represent those initially evaluated for suspected CRPS who received another chronic pain diagnosis. The grey line represents the cut-off for positive samples (mean +2 SD of healthy controls). Pairwise group comparisons were performed using Welch’s *t*-test; only statistically significant comparisons are shown, indicated by brackets and denoted by asterisks (* *p* < 0.05 and **** *p* < 0.0001). Abbreviations: M2R, muscarinic M2 receptor; β1AR, β1-adrenergic receptor; β2AR, β2-adrenergic receptor; CRPS, Complex regional pain syndrome; OCP, Other chronic pain.

Autonomic receptor autoantibody levels across study groups are summarized in Table 2 and Figure 1. M2R autoantibody levels were higher in both CRPS patients and patients with other chronic pain compared with healthy controls (CRPS vs. healthy: MD = 0.37 95%CI 0.22–0.51; other chronic pain vs. healthy: MD = 0.31 95%CI 0.19–0.44; Table 2). β2AR autoantibody levels were higher in patients with other chronic pain compared with healthy controls (MD = 0.29 95%CI 0.04–0.54), while levels in CRPS showed a comparable pattern but did not reach statistical significance (MD = 0.21 95%CI −0.01–0.42). In contrast, β1AR autoantibody levels did not differ meaningfully between pain groups and healthy controls.

**Figure 2:**
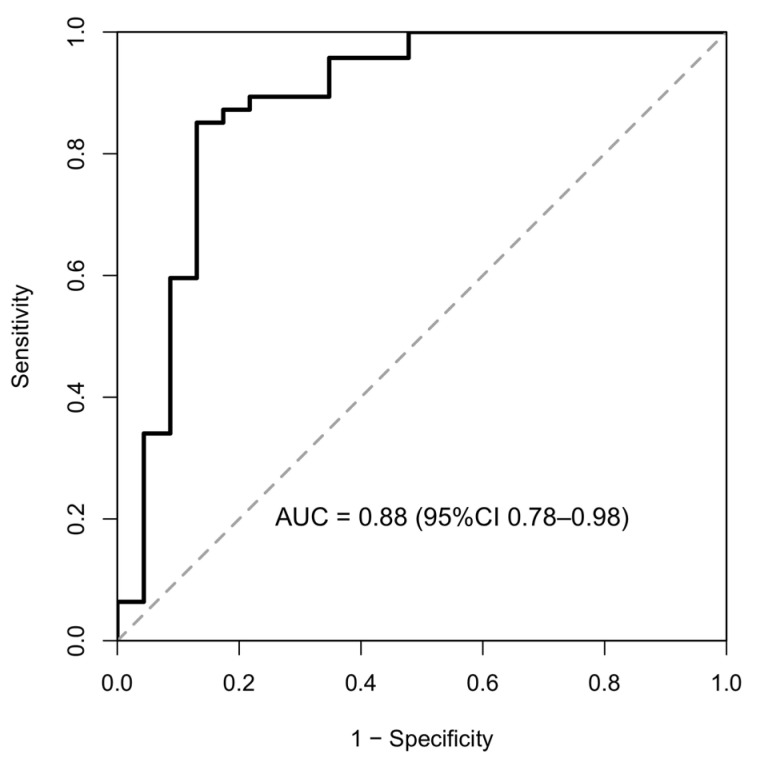
Receiver operating characteristic (ROC) curve illustrating the discrimination between chronic pain patients (CRPS and other chronic pain combined) and healthy controls based on muscarinic M2 receptor (M2R) autoantibody serum levels. The area under the curve (AUC) with 95% confidence interval is shown.

No relevant differences were observed between CRPS and other chronic pain for M2R or β2AR autoantibody levels. Exploratory ROC analysis demonstrated moderate discrimination between patients with chronic pain (CRPS and other chronic pain combined) and healthy controls based on M2R autoantibody levels (AUC = 0.88 95%CI 0.78–0.98; Figure 2). Within the CRPS group, autoantibody levels did not correlate with CSS (M2R: ρ = 0.20, *p* = 0.38; β2AR: ρ = 0.10, *p* = 0.65; β1AR: ρ = 0.12, *p* = 0.59).

Autonomic receptor autoantibody seropositivity (defined as positivity for M2R, β1AR, or β2AR) was observed in 55% (12/22) of CRPS patients, 44% (11/25) of patients with other chronic pain, and 22% (5/23) of healthy controls. This corresponds to an OR of 4.17 (95%CI 1.01–19.91; *p* = 0.03) in CRPS compared to healthy controls and OR of 2.77 (95%CI 0.69–12.69; *p* = 0.13) in other chronic pain compared to healthy controls. Seropositivity was primarily driven by M2R and β2AR autoantibodies, while β1AR seropositivity was rare (Table 3). ANA seropositivity was observed in 32% (7/22) of CRPS patients and 40% (10/25) of patients with other chronic pain and did not differ significantly between groups (Table 3). Seropositivity for either ANAs or an autonomic receptor antibody was observed in 15 CRPS patients (68%) and 18 patients with other chronic pain (72%), while co-existence of both ANA and autonomic receptor autoantibodies was observed in 4 CRPS (18%) and 3 other chronic pain patients (12%), respectively.

Results obtained with the commercially available assay (CellTrend) showed a similar pattern for β2AR autoantibody serum levels, with higher values in both pain groups compared with healthy controls (Figure S1).

## 4. Discussion

In this cross-sectional analysis of patients prospectively referred with suspected CRPS, we examined whether autonomic receptor autoantibody serum levels differentiate confirmed CRPS from other chronic pain conditions and healthy controls. We found significantly higher M2R autoantibody serum levels in both CRPS and other chronic pain compared with healthy controls. β2AR autoantibody serum levels were significantly higher in patients with other chronic pain compared with healthy controls, whereas levels in CRPS showed a similar direction but did not reach statistical significance. β1AR serum levels did not differ meaningfully between groups. Importantly, no differences were observed between CRPS and other chronic pain conditions. Together, these findings indicate that autonomic receptor autoantibody levels do not discriminate between CRPS and other chronic pain conditions and may reflect immune features associated with chronic pain more broadly.

Over the past decades, autoimmune involvement has been proposed in CRPS, although its specificity and clinical relevance remain uncertain. Early studies reported IgG reactivity against sympathetic neurons. Subsequent work identified specific targets, including M2R, β2AR, and α1A-AR, with functional effects shown in experimental systems [10,13,15,16]. In addition, increased ANA seroprevalence has been reported in a CRPS cohort (27 of 82; 33%), suggesting the presence of broader autoimmune alterations [14]. However, much of the functional evidence derives from experimental models, and clinical data remain limited.

In the present study, M2R autoantibody levels were higher in CRPS compared to healthy controls, whereas β2AR autoantibody levels did not reach statistical significance. Consistent with prior reports, we did not detect autoantibodies against β1AR in either pain group, and ANA seroprevalence was comparable to previous observations [10,14]. In contrast to prior reports demonstrating differences between CRPS and non-CRPS control groups [16], we observed no meaningful differences between CRPS and other chronic pain conditions. Taken together, these findings therefore argue against disease specificity and suggest that autonomic receptor autoantibody responses may reflect immune features associated with chronic pain more broadly.

This broader perspective aligns with emerging evidence that GPCR-targeting autoantibodies are increasingly recognized as components of physiological immune regulation and are also detectable in healthy individuals [25,26]. Among the epitopes targeted by GPCR autoantibodies, the EL2 is frequently implicated, as it is readily accessible to circulating antibodies and plays a critical role in receptor activation and ligand recognition [26,27]. Antibody binding to this domain has been linked to functional modulation of receptor signaling, including agonistic or antagonistic effects, rather than mere receptor occupancy [28]. Within this framework, many GPCR-specific autoantibodies are thought to participate in regulatory immune networks rather than functioning as passive disease markers [25]. Cross-sectional serum levels may therefore not reflect functional receptor interactions or context-dependent biological effects.

Against this background, the absence of CRPS-specific elevations in our cohort may reflect the broader, context-dependent nature of GPCR-directed autoantibody biology. In CRPS, autonomic receptor autoantibodies have been hypothesized to contribute to characteristic temperature and color asymmetries by interfering with autonomic regulation and vasomotor control [10,15]. However, the exact mechanisms and the extent to which circulating autoantibody levels relate to clinical expression remain unresolved. We also found no correlation between autoantibody levels and CSS, further limiting interpretation regarding clinical relevance. Moreover, GPCR-directed autoantibodies have been described in various conditions such as autonomic dysregulation, myalgic encephalomyelitis/chronic fatigue syndrome, and pain [22,25,29,30,31]. Such overlap, also observed in our study, may indicate a disturbance in neuroimmune homeostasis rather than distinct, condition-specific autoimmunity.

The context-dependent effects highlight the complexity of GPCR autoantibody biology and frame important considerations for interpreting the present findings. First, the cross-sectional design and single-timepoint sampling do not allow inference regarding temporal relationships or direct pathogenic mechanisms. This is particularly relevant given fluctuations in the clinical presentation of CRPS [3], and the context-dependent behavior of GPCR-directed autoantibodies [26]. Transient or state-dependent effects may therefore not be reflected by absolute serum levels measured at a single timepoint. Moreover, although GCPR autoantibodies are increasingly recognized as regulatory effectors [25], no functional assays were performed. Our conclusions are therefore limited to the detection of circulating autoantibodies rather than their downstream biological activity. Accordingly, the findings should be interpreted as exploratory and hypothesis-generating. Third, β2AR autoantibody measurements were performed using two different ELISA formats, which are not directly comparable due to differences in epitope exposure and conformational sensitivity. In particular, the peptide-based ELISA selectively detects antibody reactivity against the isolated EL2, rather than presenting the full extracellular architecture of the receptor. Although EL2 constitutes a conformationally dynamic region of β2AR, suggesting that linear peptides may preserve certain native epitope characteristics, this assay cannot fully recapitulate antibody recognition of conformational or discontinuous epitopes present on the intact receptor in vivo [26,28]. As a consequence, peptide-based measurements may underestimate or differentially capture functionally relevant antibody populations. Nonetheless, the consistent directionality of the findings across both assay formats supports the robustness of the observed β2AR-associated signal. Finally, our cohort composition and sample size may have influenced the lack of CRPS specificity. Although all patients were initially evaluated for suspected CRPS, the underlying diagnoses and mechanisms in the other chronic pain group were heterogeneous. Moreover, CRPS itself is clinically heterogeneous in terms of disease duration, clinical subtype, and treatment exposure. Our cohort predominantly comprised patients with persistent disease, and it cannot be excluded that immune alterations differ in acute CRPS. Disease stage may therefore influence the detection of potential between-group differences. While stratified analyses could help address these sources of variability, the present study was not powered for such analyses. The relatively small group sizes also limit the ability to exclude modest between-group effects. These factors may therefore limit generalizability. Nonetheless, the similar pattern across different receptor targets is consistent with a broader immunological signal across chronic pain conditions rather than a CRPS-specific autoantibody profile.

## 5. Conclusions

Autonomic receptor autoantibodies were observed across chronic pain conditions but were not specific to CRPS. These findings are consistent with broader immune alterations associated with chronic pain rather than a disease-specific autoantibody signature. Further longitudinal and functional studies are required to clarify the temporal dynamics and potential biological relevance of these autoantibodies in pain conditions.

## Figures and Tables

**Table 1:** Baseline demographic and clinical characteristics.

Characteristics	CRPS (n = 22)	Other Chronic Pain ^a^ (n = 25)	Healthy Controls (n = 23)
Age in years, median (IQR)	43 (30–54)	43 (29–60)	53 (36–58)
Sex, female, n (%)	18 (82)	17 (68)	15 (62)
Disease duration in months, median (IQR)	26 (16–74)	20 (10–36)	-
Precipitating injury, n (%)			
Trauma	10 (45)	11 (44)	-
Operation	9 (41)	10 (40)	-
Spontaneous/unknown	3 (14)	4 (16)	-
Affected limb, n (%)			
Upper right	4 (18)	5 (20)	-
Upper left	4 (18)	5 (20)	-
Lower right	4 (18)	6 (24)	-
Lower left	10 (45)	9 (36)	-
Current NRS, median (IQR)	7 (6–8)	7 (3–8)	-
Average NRS past 24 h, median (IQR)	8 (7–8)	8 (7–8)	-
CRPS severity score, median (IQR)	11 (10–12)	-	-

^a^ The other chronic pain group consisted of 14 cases of myofascial pain syndrome, 6 cases of neuropathic pain, 2 cases of inflammation, and 3 cases of unknown or unclear etiology. Abbreviations: CRPS, Complex regional pain syndrome; NRS, Numeric rating scale.

**Table 2:** Autonomic receptor autoantibody levels in patients with CRPS, other chronic pain, and healthy controls.

Marker	Group	n	Mean ± SD	Mean Difference (95%CI)	*p*-Value
M2R	Healthy controls	23	0.29 ± 0.22	Ref	–
	Other chronic pain	25	0.60 ± 0.21	0.31 (0.19–0.44)	<0.001
	CRPS	22	0.66 ± 0.25	0.37 (0.22–0.51)	<0.001
β2AR	Healthy controls	23	0.57 ± 0.42	Ref	–
	Other chronic pain	25	0.86 ± 0.44	0.29 (0.04–0.54)	0.02
	CRPS	22	0.78 ± 0.29	0.21 (−0.01–0.42)	0.06
β1AR	Healthy controls	23	0.61 ± 0.48	Ref	–
	Other chronic pain	25	0.65 ± 0.39	0.04 (−0.29–0.22)	0.78
	CRPS	22	0.57 ± 0.27	−0.04 (−0.20–0.27)	0.76

Mean differences (MD) with 95% confidence intervals (CI) and *p*-values are shown for comparisons versus healthy controls using Welch’s *t*-tests. Abbreviations: M2R, muscarinic M2 receptor; β1AR, β1-adrenergic receptor; β2AR, β2-adrenergic receptor.

**Table 3:** Frequency of ANAs and autonomic receptor autoantibody positivity in patients and controls.

Characteristics	CRPS (n = 22)	Other Chronic Pain (n = 25)	Healthy Controls (n = 23)	*p*-Value ^a^
ANAs ^b^, n (%)	7 (32)	10 (40)	-	0.78
M2R, n (%)	9 (41)	7 (28)	1 (4)	<0.01
β1AR, n (%)	0 (0)	0 (0)	2 (9)	0.20
β2AR, n (%)	8 (36)	9 (36)	2 (9)	0.04
Any positive autoantibody ^c^, n (%)	12 (55)	11 (44)	5 (22)	0.07
Coexisting M2R and β2AR, n (%)	5 (23)	5 (20)	0 (0)	0.04
Coexisting ANA and any autoantibody, n (%)	4 (18)	3 (12)	-	0.16
Coexisting ANA or any autoantibody, n (%)	15 (68)	18 (72)	-	0.35

^a^ *p*-values were calculated using the Chi-squared test or Fisher’s exact test (where appropriate) for categorical variables. ^b^ ANAs were assessed in patients only and not compared with healthy controls. ^c^ Any positive autoantibody of either the M2R, β1AR or β2AR. The corresponding OR was 4.17 (95%CI 1.01–19.91; *p* = 0.03) in CRPS compared to healthy controls and OR 2.77 (95%CI 0.69–12.69; *p* = 0.13) in other chronic pain compared to healthy controls. Abbreviations: ANAs, Anti-nuclear antibodies; M2R, muscarinic M2 receptor; β1AR, β1-adrenergic receptor; β2AR, β2-adrenergic receptor.

## Data Availability

The data that support the findings of this study are available from the Erasmus MC University Medical Center, but restrictions apply to the availability of these data, which were used under license for the current study, and so are not publicly available. Data are, however, available from the authors upon reasonable request and with permission of the Erasmus MC University Medical Center.

## Supplementary Materials

The following supporting information can be downloaded at: https://www.mdpi.com/article/10.3390/biomedicines14040945/s1, Figure S1: Autoantibody levels against the β2AR in serum from healthy controls, patients with other chronic pain, and patients with CRPS. Positive and suspected positive thresholds are indicated by the solid and dashed grey lines, respectively, based on the manufacturer’s protocol. Brackets denote pairwise group comparisons (Kruskal-Wallis test), with significance indicated by asterisks (ns = non-significant, * *p* < 0.05, ** *p* < 0.01). β2AR = β2-adrenergic receptor, CRPS = Complex regional pain syndrome, Other chronic pain = patients initially evaluated for suspected CRPS who received another chronic pain diagnosis.

## Abbreviations

The following abbreviations are used in this manuscript:

CRPS	Complex regional pain syndrome
ANAs	Antinuclear antibodies
GPCRs	G protein-coupled receptors
α1A-AR	α-1A-adrenergic receptor
β1AR	β1-adrenergic receptor
β2AR	β2-adrenergic receptor
M2R	Muscarinic M2 receptor
TMB	3,3′,5,5′-Tetramethylbenzidine
ELISA	Enzyme-linked immunosorbent assay
SD	Standard deviation
IQR	Interquartile range
MD	Mean difference
CI	Confidence interval
ROC	Receiver operating characteristic
CSS	CRPS severity score
OD	Optical density
OR	Odds ratio
