# Supplementary material for: Autonomic Receptor Autoantibodies in Complex Regional Pain Syndrome and Other Chronic Pain Conditions: A Cross-Sectional Analysis
Source: Biomedicines. 2026 Apr 21;14(4):945. doi: 10.3390/biomedicines14040945 (PMC13113101; doi:10.3390/biomedicines14040945)
Supplement: Supplementary file 1 [file biomedicines-14-00945-s001.zip › biomedicines-4254769-supplementary.pdf]

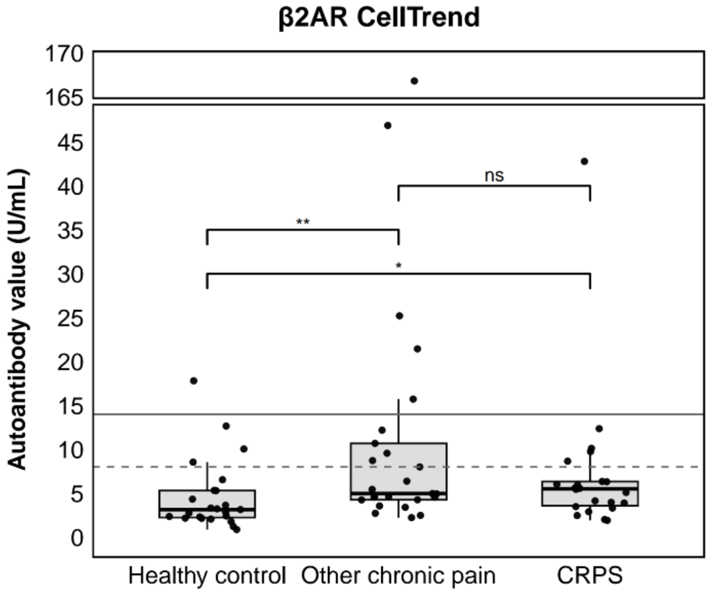

Supplementary Figure S1. Autoantibody levels against the β2AR in serum from healthy controls, patients with other chronic pain, and patients with CRPS. Positive and suspected positive thresholds are indicated by the solid and dashed grey lines, respectively, based on the manufacturer's protocol. Brackets denote pairwise group comparisons (Kruskal-Wallis test), with significance indicated by asterisks (ns= non-significant, * $p < 0.05$ ** $p < 0.01$). β2AR = β2-adrenergic receptor, CRPS= Complex regional pain syndrome, Other chronic pain = patients initially evaluated for suspected CRPS who received another chronic pain diagnosis.
